# Supplementary material for: Photo-thermionic effect in vertical graphene heterostructures
Source: Nat Commun. 2016 Jul 14;7:12174. doi: 10.1038/ncomms12174 (PMC4947168; doi:10.1038/ncomms12174)
Supplement: Supplementary Information — Supplementary Figures 1-7, Supplementary Notes 1-7 and Supplementary References [file ncomms12174-s1.pdf]

Supplementary Figures

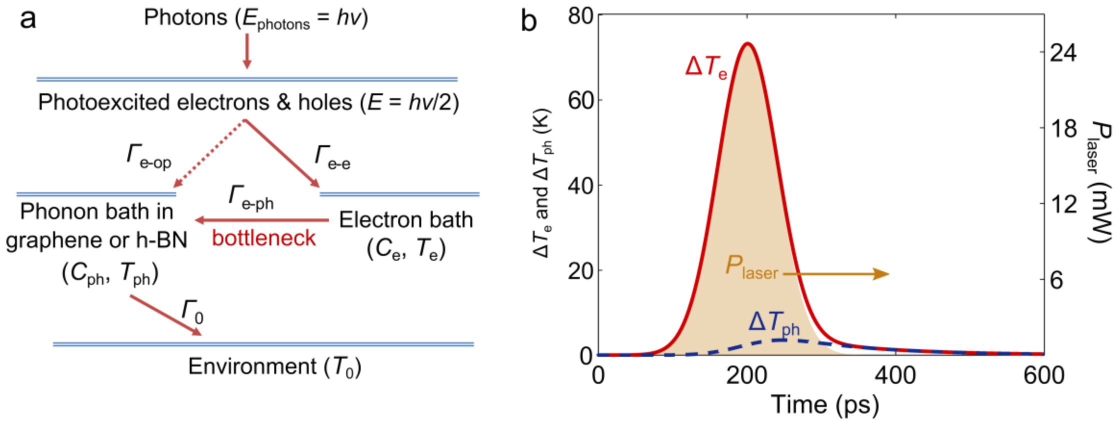

Supplementary Figure 1: Heating and cooling pathways of hot carriers in graphene. a) Schematics illustrating the cooling pathway of photoexcited carriers. Detailed descriptions are provided in the text of Supplementary Note 1. **b)** Time dependence of the rise in electron ($\Delta T_e = T_e - T_0$, red solid line) and phonon ($\Delta T_{ph} = T_{ph} - T_0$, blue dotted line) temperature calculated with the model illustrated in **a** under a quasi-CW pulse (full width at half maximum (FWHM) duration dt = 100 ps, average laser power P = 100 μW and repetition rate f = 40 MHz) at T_0 = 300K, graphene Fermi level μ = 0.2 eV. The pale yellow area represents the instantaneous laser power P_{laser} of a single pulse centered at 200 ps.

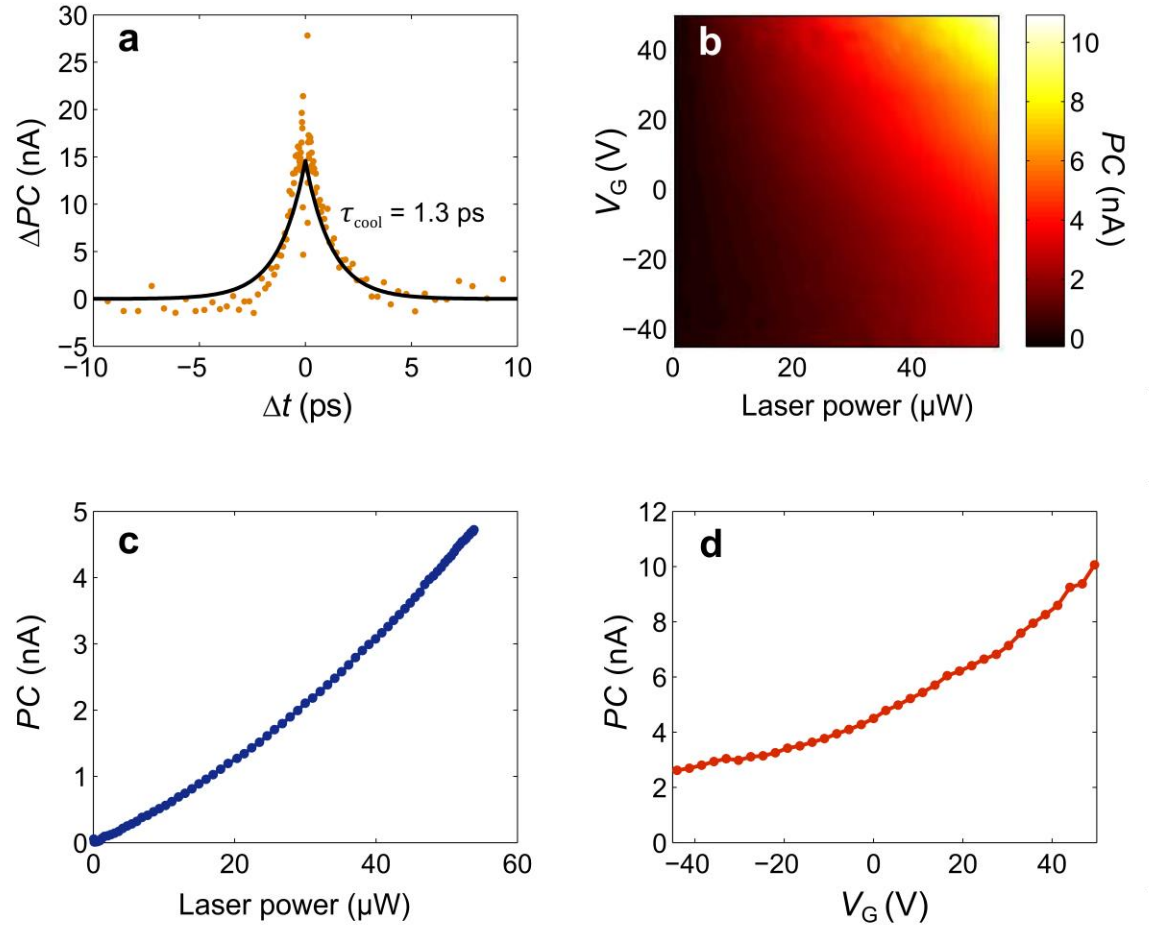

Supplementary Figure 2: PTI photocurrent in G/2.2-nm-thick WSe_2/G heterostructure. a) Photocurrent autocorrelation measurement performed with an average laser power P = 600 μW at T_0 = 30 K and bias voltage V_B = 0.5 V. The decay of $\Delta PC = PC(\Delta t) - PC(\Delta t \rightarrow \infty)$ is fitted with an exponential with time constant τ_{cool} =1.3 ± 0.1 ps (black solid line). **b)** *PC* vs. laser power and gate voltage V_G measured at bias voltage V_B = 0.7 V and T_0 = 30 K, with laser wavelength λ = 1300 nm. **c**) *PC* vs. laser power for V_G = 0 V and (**d**) *PC* vs. V_G for P = 50 μW taken from (**b**).

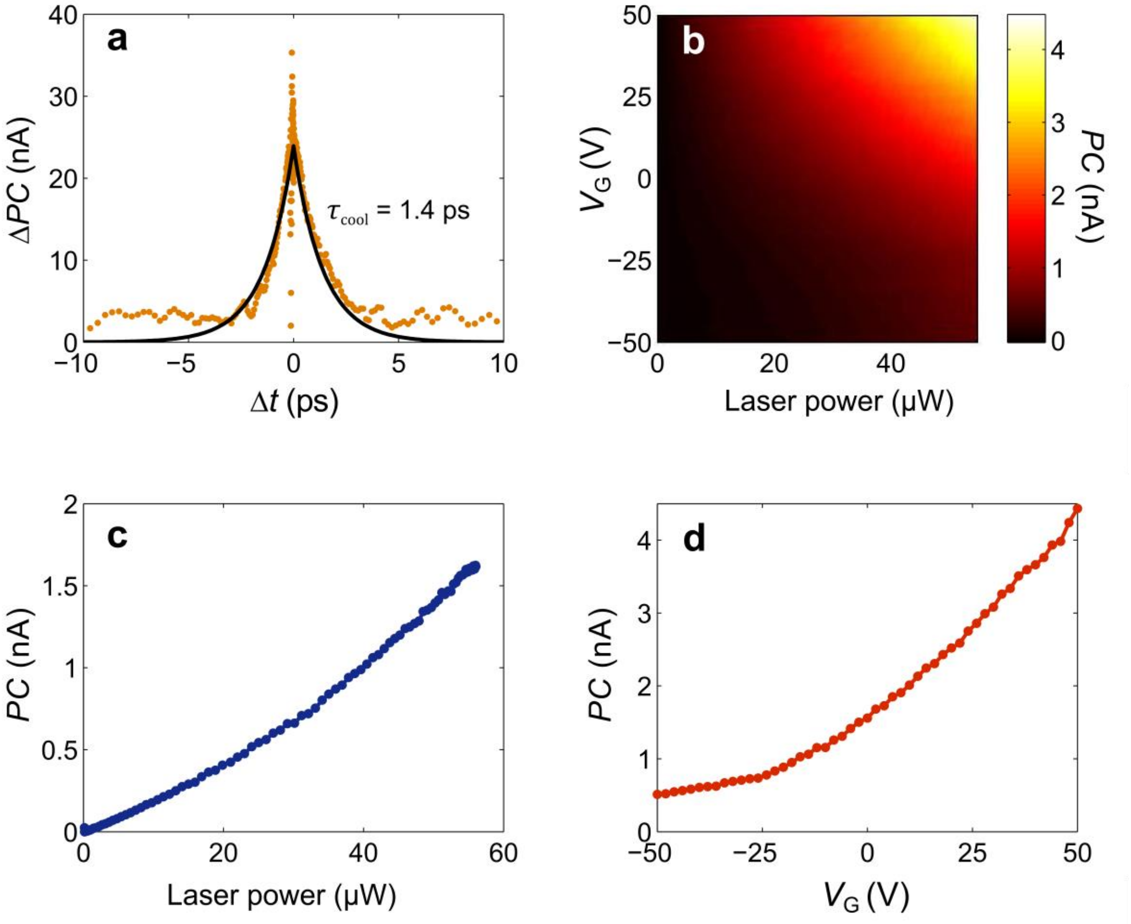

Supplementary Figure 3: PTI photocurrent in G/7.4-nm-thick WSe2/G heterostructure. a) Photocurrent autocorrelation measurement performed with an average laser power P = 825 μW at T_0 = 300 K and bias voltage V_B = 0.06 V. The decay of $\Delta PC = PC(\Delta t) - PC(\Delta t \rightarrow \infty)$ is fitted with an exponential with time constant τ_{cool} =1.4 ± 0.1 ps (black solid line). **b)** PC vs. laser power and gate voltage V_G measured at V_B = 0.5 V and T_0 = 35 K, with laser wavelength λ = 1300 nm. **c**) PC vs. laser power for V_G = 0 V and (**d**) PC vs. V_G for P = 50 μW taken from (**b**).

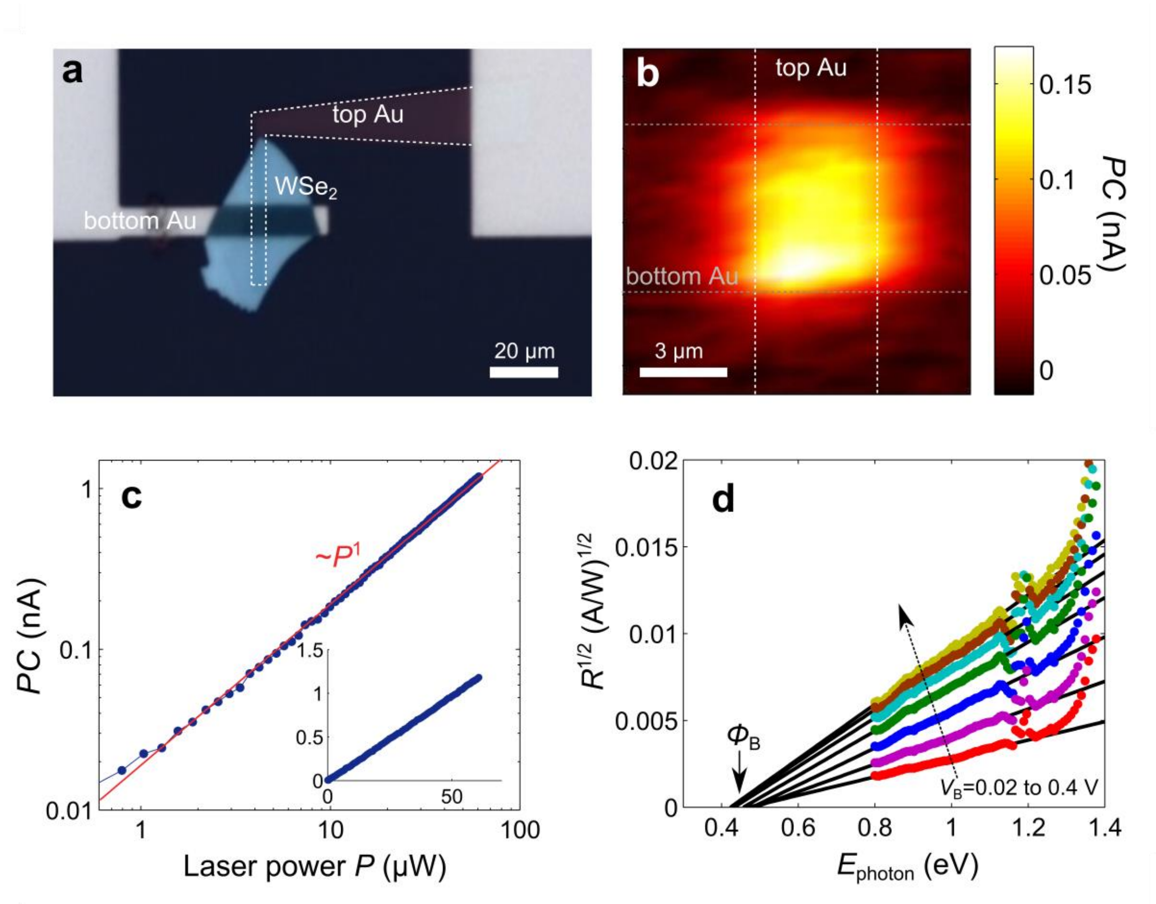

Supplementary Figure 4: Photocurrent measurements in Au/20-nm-thick WSe_2/Au heterostructure. a) Optical image of the device. **b)** Photocurrent map performed at T_0 = 300 K and V_B = 0.2 V, with a laser wavelength λ = 1500 nm and power P = 10 μW. The position of the top and bottom Au electrodes is indicated by the white and gray dotted lines, respectively. **c)** Log-log plot of PC vs. laser power measured at T_0 = 300 K and V_B = 0.2 V, with λ = 1500 nm. The red solid line corresponds to a linear power dependence. Inset: same data on linear scale. **d)** Square root of the responsivity R vs. photon energy E_{photon} at bias voltage V_B from 0.02 V (red) to 0.4 V (yellow). The black solid lines are linear fits to the data.

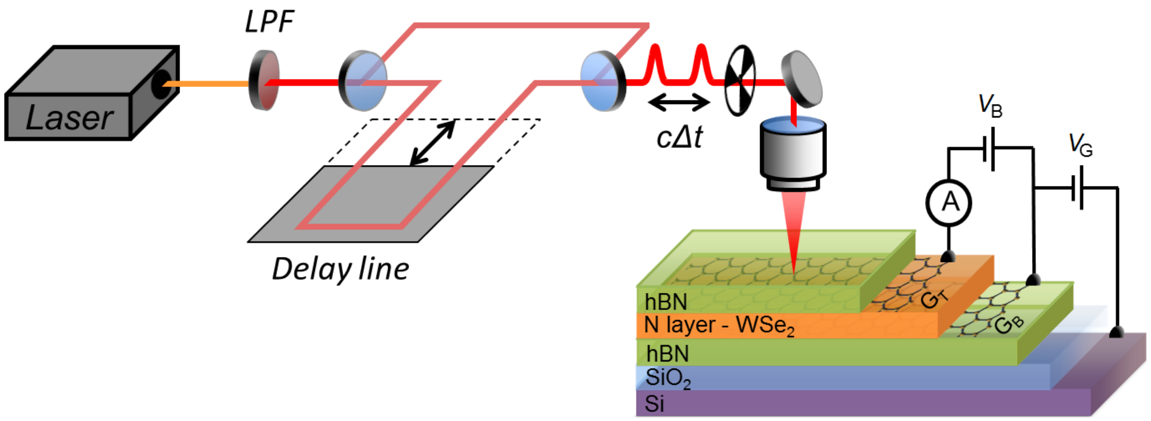

Supplementary Figure 5: Time-resolved photocurrent measurement setup. A Ti:sapphire laser generates ultrashort and broadband pulses which can be spatially delayed using a motorized delay stage by a distance *cΔt* where *c* is the speed of light. A 800-nm long pass filter (LPF) is inserted to improve the PTI signal.

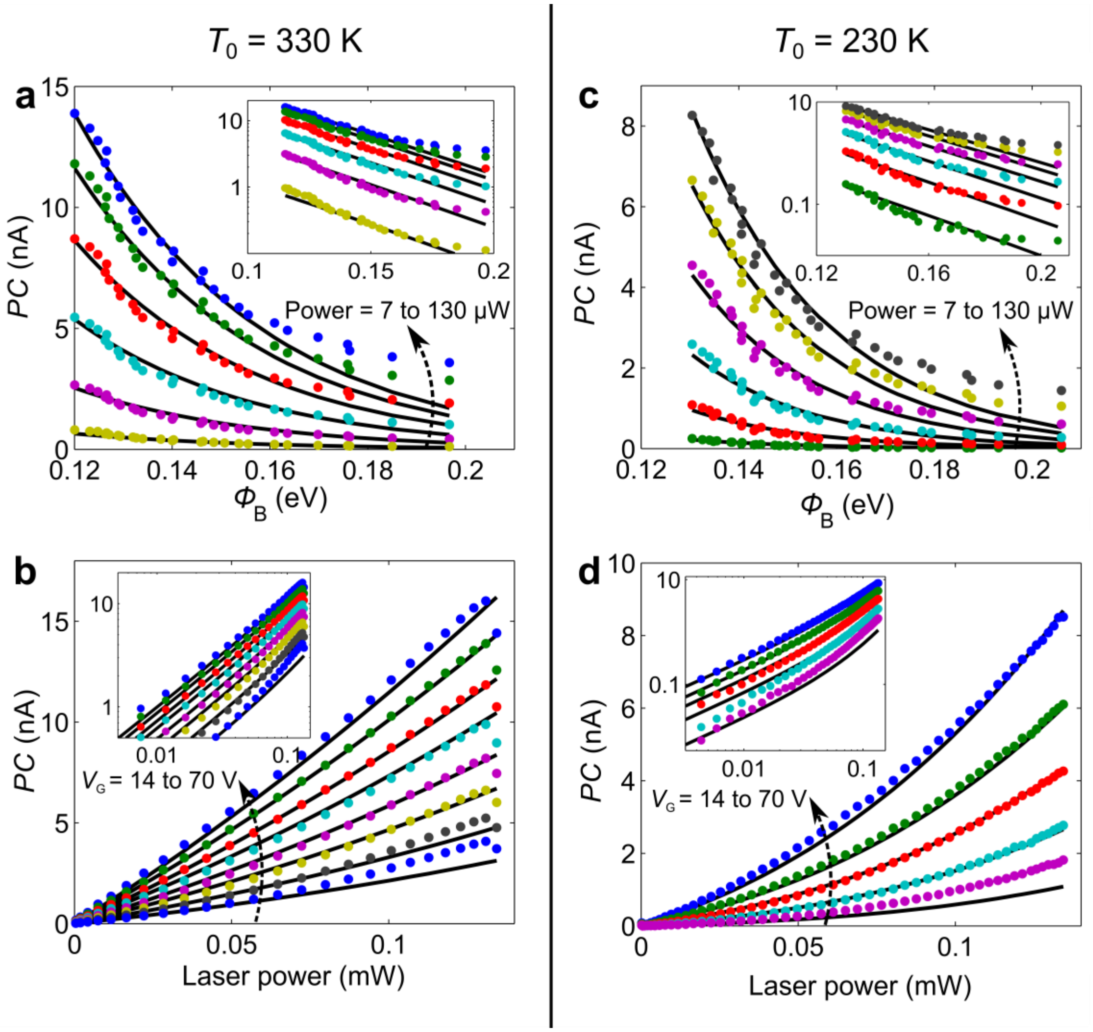

Supplementary Figure 6: Comparison between the PTI model and the experimental photoresponse measured at T_0 = 230 and 330 K, with λ = 1500 nm and V_B = 0.36 V in a G/28-nm-thick WSe_2/G heterostructure. a,c) *PC* vs. Φ_B at various laser powers and (**b,d**) *PC* vs. laser power *P* at different gate voltages V_G measured at (**a,b**) T_0 = 330 K and (**c,d**) T_0 = 230 K. The data points correspond to the experiment and the solid lines to the model. Insets: Same experimental data and theoretical curves in logarithmic scale.

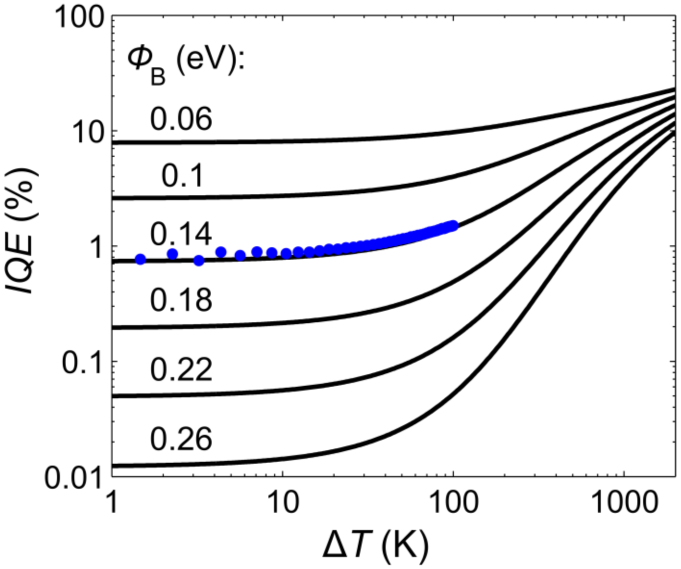

Supplementary Figure 7: Internal Quantum Efficiency (*IQE*) vs. laser-induced temperature change *ΔT*. The solid lines are theoretical *IQE* calculated with the PTI model (see Method section of main text) with τ_{inj} = 47 ps and Γ = 0.5 MWm^{-2}K^{-1}, at T_0 = 300 K and at different values of Φ_B (indicated on top of each line). The data points are taken from the measurements presented in Figure 4b of the main text, at Φ = 0.14 eV.

Supplementary Note 1: Cooling pathways of thermalized hot carriers in graphene

The heating and cooling of charge carriers in graphene is the subject of intense investigation and many different energy relaxation mechanisms have been suggested. For our experiment, we consider the energy pathways illustrated in Supplementary Figure 1a. Photons with an energy larger than twice the graphene Fermi level ($E_{photon} > 2\mu$) are absorbed in graphene due to interband transitions, creating photoexcited electrons and holes with energy $E = E_{photon}/2$. This energy is transferred to the phonon and electron bath typically through optical phonon emissions or carrier-carrier collisions, respectively. These processes are characterized by the energy-loss rate of the photoexcited carrier due to optical phonon emission $\Gamma_{e\text{-}op}$ and carrier-carrier scattering $\Gamma_{e\text{-}e}$ (Supplementary Figure 1a). Due to strong carrier-carrier interactions in graphene, carriers thermalize among themselves on ultrafast timescale (within 50 fs)[1], which leads to a branching ratio[2]

between the two processes $\Gamma_{e\text{-}e}/\Gamma_{e\text{-}op}$ larger than 1. This implies that most of the absorbed photon energy is redistributed to the electron bath. Hence, in our experiment we consider that $\eta_{heat} = 70\%$ of the laser power absorbed in graphene is transferred to the electron bath[2], giving rise to a thermalized hot carrier distribution with temperature T_e.

Hot thermalized carriers subsequently cool down to equilibrate with the phonon (lattice) temperature T_{ph} and the ambient temperature T_0. This cooling can be due to various processes such as the emission of intrinsic acoustic phonons[3], disorder-enhanced supercollisions with acoustic phonons[4,5], interaction with remote surface polar phonon modes (SPP) of the substrate[6] and in-plane heat dissipation via diffusion of hot carriers. All these mechanisms exhibit a different dependence on T_e. To simplify, we assume that for low increase in electronic temperature ($\Delta T = T_e - T_{ph} << T_{ph}$) the cooling of hot carriers is proportional to ΔT with an electron-phonon coupling constant $\Gamma_{e\text{-}ph}$. This coupling increases the phonon bath temperature T_{ph} and is finally dissipated through the substrate (at temperature T_0) at a rate Γ_0. Hence, we model the temperatures of the electron and phonon bath using the following equations:

$$C_e \frac{\partial T_e}{\partial t} = P_{in}(t) - \Gamma_{e-ph}(T_e - T_{ph}) \qquad \text{(Supplementary Equation 1)}$$

$$C_{ph} \frac{\partial T_{ph}}{\partial t} = \Gamma_{e-ph}\left(T_e - T_{ph}\right) - \Gamma_0(T_{ph} - T_0) \qquad \text{(Supplementary Equation 2)}$$

where C_e is the electronic heat capacity defined as the product of the 2D Sommerfeld constant and the electron temperature, $C_e = \gamma T_e = (2\pi\mu k_B^2/3\hbar^2 v_F^2)T_e$. Here, k_B is the Boltzmann's constant, v_F is graphene's Fermi velocity and $\hbar$ is the reduced Planck constant. $P_{in}(t)$ is the laser power density that is absorbed by graphene (approximately 0.5% absorption considering the dielectric permittivity of the surrounding medium[7]) and transferred to the electronic bath ($\eta_{heat} = 70\%$) (Supplementary ref. 2). C_{ph} is the phonon heat capacity which we assume to be roughly 10^4 times larger than C_e (Supplementary ref. 8).

The temperature dynamics predicted by supplementary equations 1 and 2 largely depends on the rate-limiting relaxation step. Since the out-of-plane (c-axis) thermal conductivity of boron nitride[9] is approximately 2 $Wm^{-1}K^{-1}$, we estimate Γ_0 ~ 30 $MWm^{-2}K^{-1}$ for our 70-nm-thick hBN substrate. This value is much larger than theoretical estimates[10] of $\Gamma_{e\text{-}ph}$, which are typically between 0.5 and 5 $MWm^{-2}K^{-1}$. Therefore, the electron-phonon cooling creates a "bottleneck" that confines the heat in the electron bath. We also note that since the in-plane electronic thermal conductivity κ_e is small (κ_e ~ 1 $Wm^{-1}K^{-1}$), the cooling due to lateral diffusion of hot carriers ($\sim h\kappa_e/A$, where h = 0.3 nm is the thickness of graphene and A = 2.5 μm^2 is the laser spot size) is negligible (i.e., $\Gamma_{e\text{-}ph} >> h\kappa_e/A$) (Supplementary ref. 10).

For steady-state conditions and $\Gamma_{e\text{-}ph} << \Gamma_0$, Supplementary Equations 1 and 2 simplify to

$T_\mathrm{e} - T_0 \cong P_\mathrm{in}/\Gamma_\mathrm{e-ph}$ (Supplementary Equation 3)

We use this relation to model the PTI effect in the main text (see Methods) and find that the data is best described with $\Gamma_{e\text{-}ph}$ = 0.5 ± 0.3 $MWm^{-2}K^{-1}$. This value is compatible with the calculated out-of-plane thermal conductance of G/hBN interfaces caused by electron coupling with SPP phonons[6]. It is also consistent with a disorder-enhanced supercollision cooling mechanism[11] which predicts $\Gamma_\mathrm{e-ph} \cong 3\Sigma T_\mathrm{ph}^2$ under steady-state conditions[5], where Σ is the supercollision rate coefficient (typically between 0.5 and 2 $Wm^{-2}K^{-3}$) (Supplementary ref. 4).

To verify the validity of Supplementary Equation 3 for our experimental conditions, we solve Supplementary Equations 1 and 2 to exactly calculate the variation in temperature of the electron ($\Delta T_e = T_e - T_0$) and phonon ($\Delta T_{ph} = T_{ph} - T_0$) baths induced by a quasi-CW laser pulse similar to the one used in our experiment (pulse duration dt = 100 ps and repetition rate f = 40 MHz) with average laser power P = 100 μW, at T_0 =300 K and using $\Gamma_{e\text{-}ph}$ = 0.5 $MWm^{-2}K^{-1}$ and Γ_0 = 30 $MWm^{-2}K^{-1}$. The calculations (Supplementary Figure 1b) show that the T_e reaches a temperature that is much higher than T_{ph} (and T_0) and in quantitative agreement with Supplementary

Equation 3. Moreover, the temperature closely follows the variation of the pulse intensity in time. This confirms the validity of the steady-state approximation, which is expected since the measured cooling time τ_{cool} is much shorter than the pulse duration. More importantly, these calculations indicate that the electron bath is thermally decoupled from the phonon bath ($T_e > T_{ph}$) under (quasi-) steady-state conditions.

Supplementary Note 2: Devices with different WSe_2 thicknesses

We have varied the thickness L of the WSe_2 layer providing the energy barrier between the two graphene sheets. All the data shown in the main text come from a device containing WSe_2 layer with L = 28 nm. We also measured sub-bandgap photocurrent on devices with L = 2.2, 7.4 and 55 nm. These devices are made using the layer assembly technique described in Supplementary ref. 12, and are deposited on a Si/SiO_2 substrate that acts as a gate electrode. All devices display features in photocurrent that we attribute to the PTI effect. Supplementary Figures 2a and 3a show positive photocurrent autocorrelation peaks in devices with L = 2.2 and 7.4 nm (for details on the measurement technique see Supplementary Note 4). As discussed in the main text, the dynamics of this peak is characterized by a time constant $\tau_{cool} \sim$ 1-2 ps which is on the order of the cooling time of the hot carriers in graphene. We also observe a superlinear power dependence of the photocurrent in these devices (Supplementary Figures 2c and 3c), which is characteristic of the PTI effect. Finally, the increase in photocurrent with gate voltage V_G (Supplementary Figures 2d and 3d) is consistent with the PTI effect, which depends exponentially on the Schottky barrier height Φ_B. This effect is also observed in the device with L = 55 nm. Importantly, we note that the magnitude of the photocurrent does not vary significantly with the WSe_2 thickness L, indicating that tunneling effects (which depend exponentially on L) do not likely play a role in the photocurrent generation process. These observations further reinforce the conclusion that the PTI effect

governs the photocurrent response of these heterostructures in this photon energy range.

Supplementary Note 3: Internal photoemission in Au/WSe_2/Au heterostructures

In order to emphasize the difference between the internal photoemission (IPE) process typically observed at metal/semiconductor interface and the photo-thermionic (PTI) effect measured at graphene/WSe_2 junctions, we study the sub-bandgap photocurrent generated in Au/WSe_2/Au vertical heterostructures. Supplementary Figure 4a shows such a device made with a 20-nm-thick WSe_2 layer and a 10-nm-thick Au top electrode (similar measurements were obtained on a Au/40-nm-thick WSe_2/Au device). The photocurrent map in Supplementary Figure 4b shows that photocurrent is generated by sub-bandgap photons (λ = 1500 nm) in the region where all three layers overlap. Interestingly, the magnitude of this photocurrent scales linearly with the laser power (Supplementary Figure 4c), regardless of the bias voltage V_B. This observation is consistent with the IPE process, which predicts that the number of carriers emitted over the barrier scales linearly with the number of initial photoexcited carriers. Moreover, Supplementary Figure 4d shows that the measured responsivity ($R = PC/P$) satisfies the relation $R \propto (E_{\mathrm{photon}} - \phi_{\mathrm{B}})^2$ expected for the IPE process[13]. By projecting this relation to lower E_{photon}, we find a cut-off energy at $E_{\mathrm{photon}} = \Phi_{\mathrm{B}} \approx 0.4 – 0.5$ eV. We also note that the IPE responsivity of the Au/WSe_2/Au is smaller than the PTI responsivity measured in G/WSe_2/G, especially at low photon energy. For instance, at E_{photon} = 0.8 eV, the maximum responsivity measured in Au-based device is R = 0.036 mAW^{-1}, whereas graphene-based devices can easily reach R = 0.12 mAW^{-1}.

All these observations that we attribute to IPE (i.e., linear power dependence, strong dependence on E_{photon} and cut-off at Φ_{B}) clearly contrast with the main features of the PTI effects (superlinear power dependence and no dependence on E_{photon}). This raises the question of why IPE dominates the photoresponse of metal/WSe_2 while PTI governs the one of graphene/WSe_2. The answer is two-fold. First, as discussed

in the main text, carriers thermalize among themselves much more rapidly in graphene (approximately 10 fs) than in metal (approximately 100 fs) due to the stronger carrier-carrier interaction. This ultrafast thermalization process competes directly with the internal emission of initially photoexcited carriers (IPE). Assuming the timescale of this process to be equal for graphene and metal, we can conclude that IPE in graphene is suppressed by an order of magnitude compare to metal.

Secondly, we can explain why the PTI effect in graphene is larger than in metal from the result of the PTI model presented in the main text. Indeed, under steady-state conditions (e.i. when the pulse duration is much longer than τ_{cool}, which is the case in our experiment except for time-resolved measurements), we can estimate the rise in electronic temperature as $\Delta T = P_{in}/\Gamma$, where P_{in} is the incident power delivered to the carriers and Γ is a thermal conductance term which describe the rate-limiting heat dissipation mechanism from the electronic system to the phonon bath (see Supplementary Note 1). From the fit of our PTI model we obtain $\Gamma_G = 0.5$ MWm^{-2}K^{-1} for graphene/WSe_2, while the electron-phonon coupling constant of Au, for instance, is $\Gamma_{Au} = 2.6 \times 10^{16}$ Wm^{-3}K^{-1} (Supplementary ref. 14). Considering the 10-nm-thick Au electrode of the device shown in Supplementary Figure 4a, this value corresponds roughly to a two-dimensional $\Gamma_{Au,2D}$ of 260 MWm^{-2}K^{-1}, which is two orders of magnitude larger than Γ_G. We conclude that for a given power P_{in}, the rise in electronic temperature ΔT is approximately 500 times larger in graphene than in gold (metals). This enhanced ΔT, along with the suppression of the IPE due to ultrafast thermalization of carriers in graphene, explains why the PTI effect, which depends exponentially on the carrier temperature, dominates the photoresponse of graphene/semiconductors junctions but not the one of metal/semiconductors interfaces.

Supplementary Note 4: Time-resolved photocurent measurements

Time-resolved photocurrent measurements are performed using a setup similar to the one described in Supplementary ref. 12 and shown in Supplementary Figure 5.

Pulses with a duration of approximately 200 fs and a spectral bandwidth of 200 nm centered at 800 nm are generated by a Ti:sapphire laser (Thorlabs Octavius) with a repetition rate of 85 MHz. Due to the broad emission spectrum of the laser, both photocurrent mechanisms (i.e. PTI due to graphene absorption and photoexcited charge transfer due to WSe_2 absorption) can contribute to the measured photocurrent autocorrelation signal. In order to isolate the PTI photocurrent and reduce the one originating from WSe_2 absorption, we insert an 800-nm long pass filter in the beam path. The filtered beam is split into two arms (one with a motorized delay stage) and recombined using 50/50 beam splitters. Time-resolved photocurrent measurements are performed by measuring the photocurrent (with a preamplifier and a lock-in amplifier synchronized with a mechanical chopper at 117 Hz) as a function of the time delay Δt between the laser pulses of each arm.

We can further separate the contribution of the PTI signal (originating from graphene absorption) and the one of stemming from direct WSe_2 absorption by taking advantage of their different power dependence: the superlinear (sublinear) power dependence of graphene (WSe_2) absorption leads to a positive (negative) correlation signal[12]. Furthermore, under the right conditions (typically low temperature and bias voltage), the WSe_2 photocurrent can be suppressed and the signal indeed displays a positive peak around $\Delta t = 0$ (see Figure 2d of main text, as well as Supplementary Figure 2a and 3a).

Supplementary Note 5: PTI model and measurements at different ambient temperatures

To validate our model of the PTI effect, we repeat the measurement and analysis of PC vs. laser power P and gate voltage V_G at other ambient temperatures. At $T_0 = 330$ K (Supplementary Figures 6a and b), the fit of the model gives a carrier injection time $\tau_{\mathrm{inj}} = 34 \pm 10$ ps and an out-of-plane interfacial thermal conductance $\Gamma = 1 \pm 0.6$ $\mathrm{MWm^{-2}K^{-1}}$, whereas at $T_0 = 230$ K (Supplementary Figures 6c and d), we obtain $\tau_{\mathrm{inj}} = 24 \pm 10$ ps and $\Gamma = 0.6 \pm 0.3$ $\mathrm{MWm^{-2}K^{-1}}$. We find a good agreement between the

model and experiment for both temperatures, but a more detailed study is needed in order to examine and understand the possible dependence of τ_{inj} and Γ on temperature T_0.

Supplementary Note 6: Estimation of the Internal Quantum Efficiency

Using the PTI model described in the main text, we can estimate the performance of the G/WSe_2/G heterostructure at higher electronic temperatures and lower Schottky barriers. Supplementary Figure 7 shows the predicted internal quantum efficiency (IQE, defined as the ratio between the number of carriers collected and the number of photons absorbed by the graphene layer) as a function of the change in temperature ΔT=T_e-T_0 induced by our supercontinuum laser (see Method of the main text) at λ = 1500 nm. Assuming ~0.5% absorption[7] we find that, in those conditions, the IQE can reach up to about 20% for small Schottky barriers Φ_B (smaller than 0.14 eV). We note however that our model does not take into account a possible change in thermal conductance Γ with increasing T_e and is only valid for $k_B T_e << \Phi_B$.

Supplementary Note 7: Landauer transport model

To model the injection of hot carriers over the G/WSe_2 Schottky barrier, we consider the system shown in Figure 1b of the main text, which consists of a single G_B/WSe_2 heterojunction. We note that this system does not include the top graphene layer. Indeed, as explained in the main text, the contribution of the top graphene layer can be neglected when a positive interlayer bias V_B is applied. In these conditions, according to Landauer's transport theory[15,16], the current density J flowing through the heterojunction (considered as the resistor channel) between the graphene and WSe_2 layers can be written as:

$$J = \frac{e_0}{\tau_{\mathrm{inj}}} \int_{-\infty}^{\infty} T(E) D(E) \left(f_{\mathrm{G}}(E) - f_{\mathrm{WSe_2}}(E) \right) dE \,, \qquad \text{(Supplementary Equation 4)}$$

where e_0 is the elementary charge, τ_{inj} is the time it takes for an electron to transfer through the junction, referred here as the charge injection time, $T(E)$ is the transmission probability, and $f_{\mathrm{G}}(E)$ and $f_{\mathrm{WSe_2}}(E)$ are the Fermi-Dirac function of graphene and WSe_2, respectively. $D(E)$ is the density of states of graphene, where $\hbar$ is the reduced Planck's constant and v_{F} is the graphene Fermi velocity.

We set the charge neutrality point of graphene to $E = 0$ (as illustrated in Figure 1b of the main text) and assume the tunneling contribution to the photocurrent to be negligible (which is the case for the thick WSe_2 layer considered here) and a unity transmission for energies above the Schottky barrier. We obtain:

$D(E) = 2|E|/\pi(\hbar v_{\mathrm{F}})^2$

$f_{\mathrm{G}}(E) = 1/(e^{(E-\mu)/k_{\mathrm{B}}T}+1)$

$f_{\mathrm{WSe_2}}(E) = 1/(e^{(E-\mu+e_0V_{\mathrm{B}})/k_{\mathrm{B}}T}+1)$ (Supplementary Equation 5)

and

$$T(E) = \begin{cases} 1 \text{ for } E > \phi_0 \\ 0 \text{ for } E \leq \phi_0 \end{cases}$$

where μ is the Fermi energy of graphene, V_{B} is the voltage applied across the WSe_2 layer (see Figure 1c of the main text) and ϕ_0 is the offset between the WSe_2 conduction edge and graphene's Dirac point (see Figure 1b of the main text). In reverse bias condition (such that $f_{\mathrm{G}}(E) \gg f_{\mathrm{WSe_2}}(E)$ for $E > \phi_0$), Supplementary Equation 4 becomes:

$$J = \frac{2}{\pi}\frac{e_0}{\tau_{\mathrm{inj}}}\frac{1}{(\hbar v_{\mathrm{F}})^2}\int_{\phi_0}^{\infty}|E|f_{\mathrm{G}}(E)dE \quad \text{(Supplementary Equation 6)}$$

Supplementary Equation (6) can be solved analytically provided that $\phi_{\mathrm{B}} = \phi_0 - \mu \gg k_{\mathrm{B}}T$ and we obtain the equation of the PTI emission model presented in the Methods section of the main text:

$$J = \frac{2}{\pi}\frac{e_0}{\tau_{\mathrm{inj}}}\left(\frac{k_{\mathrm{B}}T}{\hbar v_{\mathrm{F}}}\right)^2\left(\frac{\phi_0}{k_{\mathrm{B}}T}+1\right)exp\left(\frac{-\phi_{\mathrm{B}}}{k_{\mathrm{B}}T}\right) \quad \text{(Supplementary Equation 7)}$$

Supplementary References

1. Tielrooij, K. J. *et al.* Generation of photovoltage in graphene on a femtosecond timescale through efficient carrier heating. *Nature Nanotech.* **10,** 437–443 (2015).

2. Tielrooij, K. J. *et al.* Photoexcitation cascade and multiple hot-carrier generation in graphene. *Nature Phys.* **9,** 248–252 (2013).

3. Bistritzer, R. & MacDonald, A. H. Electronic cooling in graphene. *Phys. Rev. Lett.* **102,** 206410 (2009).

4. Betz, A. C. *et al.* Supercollision cooling in undoped graphene. *Nature Phys.* **9,** 109–112 (2012).

5. Graham, M. W., Shi, S.-F., Ralph, D. C., Park, J. & McEuen, P. L. Photocurrent measurements of supercollision cooling in graphene. *Nature Phys.* **9,** 103–108 (2012).

6. Low, T., Perebeinos, V., Kim, R., Freitag, M. & Avouris, P. Cooling of photoexcited carriers in graphene by internal and substrate phonons. *Phys. Rev. B* **86,** 045413 (2012).

7. Stauber, T., Peres, N. M. R. & Geim, A. K. Optical conductivity of graphene in the visible region of the spectrum. *Phys. Rev. B* **78,** 085432 (2008).

8. Benedict, L. X., Louie, S. G. & Cohen, M. L. Heat capacity of carbon nanotubes. *Solid State Commun.* **100,** 177–180 (1996).

9. Simpson, A. & Stuckes, A. D. The Thermal Conductivity of Highly Oriented Pyrolytic Boron Nitride. *J. Phys. C Solid State Phys.* **4,** 1710–1718 (1971).

10. Freitag, M., Low, T. & Avouris, P. Increased responsivity of suspended graphene photodetectors. *Nano Lett.* **13,** 1644–1648 (2013).

11. Song, J. C. W., Reizer, M. Y. & Levitov, L. S. Disorder-Assisted Electron-Phonon Scattering and Cooling Pathways in Graphene. *Phys. Rev. Lett.* **109,** 106602 (2012).

12. Massicotte, M. *et al.* Picosecond photoresponse in van der Waals heterostructures. *Nature Nanotech.* **11,** 42–46 (2015).

13. Sze, S. M., Crowell, C. R. & Kahng, D. Photoelectric determination of the image force dielectric constant for hot electrons in Schottky barriers. *J. Appl. Phys.* **35,** 2534–2536 (1964).

14. Qiu, T. Q. & Tien, C. L. Heat Transfer Mechanisms During Short-Pulse Laser Heating of Metals. *J. Heat Transfer* **115,** 835-841 (1993).

15. Datta, S. Lessons from Nanoelectronics: a New Perspective on Transport (Wold Scientific, 2012).

16. Sinha, D. & Lee, J. U. Ideal Graphene/Silicon Schottky Junction Diodes. *Nano Lett.* **14,** 4660–4664 (2014).
